# Testing a parent support intervention to improve success of first year students at a historically-Black University (HBCU)

**DOI:** 10.3389/feduc.2025.1584908

**Published:** 2025-08-14

**Authors:** Sharron Xuanren Wang, Lawita Cheatham-Hemphill, Melissa A. Harrington

**Affiliations:** Delaware State University, Dover, DE, United States

**Keywords:** parental engagement, HBCU, college students’ success, intervention, the parental university program

## Abstract

Intergenerational education mobility is one of the key dimensions of social mobility. Educational mobility is defined as the association between parents and children’s educational attainment. Children born to parents with a college degree are more likely to graduate from college. On the other hand, first generation college students (i.e., students who have parents without a college degree) are less likely to go to college and are more likely to drop out of college compared to students with college-educated parents. Previous literature has suggested that parental involvement in higher education leads to improved student performance. Parents who did not attend college, on the other hand, might not have the knowledge to help their children navigate college. College students, especially in their freshman year, face many challenges, such as a heavier workload than is typical for high school and a distracting peer culture. At our Historically Black University, we developed a year-long communication plan targeted at parents of first-year students and aimed at boosting the educational cultural capital of parents and cultivating a supportive environment to enhance students’ educational experiences and outcomes. One of our main goals was to help retain students in the academic pipeline in majors related to Science, Technology, Engineering and Mathematics (STEM), as well as the health sciences, while also motivating students to pursue graduate school or obtain a job in the field. The program has graduated 3 cohorts of parents of first year students. Applying a mixed method approach, including an online survey method and in-depth qualitative interviews, our results indicated that parents in the Parent University program benefited from the information acquired. Details about the intervention, the implications of our findings, and the lessons learned from program implementation are discussed.

## Introduction

The educational outcomes of college students are influenced by a variety of non-academic factors, including family background such as parental educational and income level, demographic background (i.e., race and gender), and campus environment such as perceived academic and social support (Dennis, 2005; Keels, 2013; Norvilitis and Reid, 2015; Norvilitis and Reid, 2012). Much research has suggested that parental involvement is one of the best predictors of educational success, and even perceived parental involvement has positive effects on college students’ success (Ratelle et al., 2005; Mailhot and Feeney, 2017; Bartle-Haring et al., 2022). In practice, many of the elements that influence a student’s educational outcomes are difficult to impossible to change. Parental involvement, on the other hand, can be enhanced and may serve as an important instrument for improving students’ success (Wang and Sheikh-Khalil, 2013; Kranstuber et al., 2012; Collings and Eaton, 2024).

Parents from underrepresented and low socio-economic status (SES) backgrounds, like other parents, report that they want “the best” for their children’s future, and higher education is often part of parent’s aspiration, regardless of SES (Bok, 2010; Leo, 2022). Parents from middle-and high-SES backgrounds usually have the cultural capital to support their children’s educational aspirations, however, parents from underrepresented and low SES backgrounds often lack that social capital or awareness about financial and other support resources available to facilitate access to higher education (Fischer et al., 2019; Sarubbi et al., 2019; Forster and van de Werfhorst, 2019; Macaulay et al., 2023). There is a strong evidentiary basis for the effectiveness of outreach to parents of primary and early secondary school-age children in supporting college access (Bergerson, 2009; Fischer et al., 2019; Mwangi et al., 2019; Mitchall and Jaeger, 2018; Boonk et al., 2018; Şengönül, 2022; Fatimaningrum, 2022; Al-Alwan, 2014). However, fewer studies have examined the role of family support in facilitating student success after college entry, and evidence of which elements and resources are most effective is especially lacking (Dennis, 2005; Fischer et al., 2019; Roksa and Kinsley, 2019; Sax and Wartman, 2010).

At our Historically-Black University, we developed a year-long communication plan targeting parents of first year students aiming to boost parents’ educational cultural capital and cultivate a supportive environment that enhances students’ educational experiences and outcomes. Our goal was to help retain students in the academic pipeline in majors related to Science, Technology, Engineering and Mathematics (STEM) and health science, and motivate them to continue on to a doctoral program. At our institutions about 70% of undergraduate students are members of underrepresented minorities, about 33% of students are first-generation college students, and over 60% qualify for Pell grants indicating lower income parents.

We piloted the Parent University program with 33 parents during the 2021–2022 academic year. For the pilot study, all parents were given the intervention, i.e., they received information aimed to enhance their educational cultural capital. Through the pilot program, we learned what works best in terms of recruitment of parents and how to deliver the intervention to them. Following the pilot study, the Parent University program was officially implemented in the 2022–2023 academic year and 2023–204 academic year. Using a mixed method approach by applying both an online survey method and in-depth qualitative interviews, we investigated the effectiveness of the Parent University program. In this paper, we (1) describe the details of the Parent University program and its intervention, (2) discuss the recruitment process and data collection, (3) present the results from the surveys and interviews, and (4) discuss its implications.

### Literature review

Older research suggests that parental involvement in higher education leads to improved student performance (Wintre and Yaffe, 2000; Kenny, 1987). College students, especially in their freshman year, face many challenges, such as a heavier workload and a distracting peer culture. For those who move away to college, there are additional challenges including adapting to new living arrangements and making new friends. Communicating with parents might decrease students’ stress level and help them cope with these life changes. However recently, a debate has emerged as parents’ involvement in their students’ college experience can have both positive and negative impacts. While parental involvement is generally seen as beneficial, there is a growing concern about over-involvement, often referred to as helicopter parenting, which can limit students’ autonomy and hinder their development (Manuel et al., 2023). This over-involvement may extend to critiquing faculty and administrators, potentially affecting the student’s independence and growth (Smith, 2018). Because of these negative effects, some colleges even try to restrict parents’ involvement in their college students’ lives (Carney-Hall, 2008).

Nationwide, first generation college and low income students consistently drop out of college at much higher rates than middle-to upper-income students with college-educated parents (Ishitani and DesJardins, 2002). Low-income and first-generation college students face various risks that can impact their retention in college. Financial concerns, lack of understanding of university culture, and limited access to support systems are key challenges that these students encounter (Higginbotham, 2022; DeAngelo and Franke, 2016; Brown et al., 2021). A lack of understanding of university culture and financial aid needs have been identified as particular barriers to retention for first-generation students (Brown et al., 2021). Indeed, first-generation students at 4-year institutions are twice as likely to drop out of college before their second year as are students whose parents have a bachelor’s degree (Terenzini et al., 1996), and are four times as likely to drop out if their parents are low-income (Engle and Tinto, 2008). Even accounting for factors such as working full-time, financial aid status, gender, and race/ethnicity, first generation status is still a significant predictor of a student leaving before their second year (Chen and Carroll, 2005; Deangelo et al., 2011). Over the last several decades, numerous studies have identified factors related to the disparity in educational attainment for underrepresented and first generation students including academic under-preparation, discrimination, feelings of alienation, difficulty adjusting to campus culture, work and family responsibilities, financial and structural barriers, and lack of support (Ishitani and DesJardins, 2002; Ramos-Sanchez and Nichols, 2007; Lotkowski, 2004; Kalkbrenner et al., 2021; Costello et al., 2018; Gibbons et al., 2016; Holden et al., 2021). At our Historically-Black public university, large numbers of our students are at the intersection of these risk factors - being a member of a minority group, being a first-generation college student, and coming from a low-income background. Therefore, programs that provide support structures to help students succeed can have a very large impact, on both the retention and graduation rates at our institution, as well as on the number of college graduates from underrepresented and low SES groups entering the workforce.

There is an on-going cultural shift in the relationship between parents and their young-adult children, in which the two parties have an increased level of interaction and interdependency (Coburn, 2006; Wartman and Savage, 2008). Most parents of today’s traditional-aged college students maintain close ties with their children (Coburn, 2006; Sax and Weintraub, 2014), and data from the National Survey of Student Engagement (NSSE) suggest that 70% of students have a high frequency of communication with parents (NSSE, 2007). This parent–child relationship is an important form of social capital (Perna and Titus, 2005; Roksa et al., 2020). Traditionally, students have served as the bridge connecting their parents with their institution. That is, parents have learned about college programs and events mainly through their children. Compared to previous generations, parents today have more direct interactions and communications with their children’s colleges (Henning, 2007), which may support their student’s successful completion. Leveraging the relationship between parents and students to promote student engagement with faculty and staff has been explored as a strategy to enhance student success during the college years (Deutschlander, 2019).

Social capital refers to an individual’s interpersonal relationships that have productive benefits (Coleman, 1988). Social capital can, in turn, produce other forms of beneficial resources, such as cultural capital - an individual’s knowledge, experience, behaviors, and skills which are typically a product of the person’s social standing in society (Bourdieu, 1985). Parents of middle and high SES have educational cultural capital that they pass on to their children and which supports their educational success (Lapsley et al., 1990; Barsegyan and Maas, 2022). However, parents who did not attend college may lack educational cultural capital to pass on to their children regardless of how often they interact. Students from disadvantaged backgrounds (e.g., first generation college students, students belonging to racial/ethnic minority groups, etc.) are more likely to be lacking in educational cultural capital. Further educating and engaging parents from disadvantaged backgrounds about their children’s undergraduate education is a potentially powerful tool for increasing parents’ educational cultural capital, which can then be passed on to their children (Abbas et al., 2021; Jæger and Karlson, 2018). This strategy can help parents become more involved in their children’s education and facilitate students’ academic and career success.

## Intervention and program evaluation

### Recruitment and intervention

For recruitment, we initially held information sessions regarding the Parent University program at the University’s New Student Orientations. Parents of new freshmen in targeted majors (i.e., STEM and health sciences) were informed of the study and were given a flyer with a QR code they could use to learn more about the program. In addition, a postcard about the program was created for parents of STEM and health sciences majors who had participated in a New Student Orientation session and/or had submitted a housing deposit. The postcard was distributed by the University’s Integrated Marketing Office. Parents interested in being part of the program were asked to complete the online registration and the consent forms. We then randomly assigned the parents into either a control group or an intervention group. Parents in both groups received a survey before the intervention and a survey after the intervention. However, only parents in the intervention group received the intervention.

Our intervention was designed to communicate information to enhance the parents’ educational cultural capital and knowledge of university events, timelines, and policies. The interventions included five virtual forum sessions and distribution of university messages to parents. Table 1 displays the forum topics and their presenters. The forum topics included an orientation about the intervention, academic expectations of being a college student, how to utilize internships and research opportunities to prepare for the transition to the workplace, health and spiritual resources on campus, and preparations for the next semester. The forums were held via Zoom. Reminders about the forums were sent to parents via email and text messages. The forums were recorded, and links for the recordings were sent to all parents in the intervention group in case some parents were not able to attend the forum synchronously. In addition, university emails and announcements were distributed to parents via emails and text messages so that the parents in the intervention group received the most up-to-date communications from the university. For example, parents would receive information on deadlines, the last day for classes, late registration, “No-Show” or “Non-Attendance” Rosters, etc. It is the hope that parents would communicate this information to their child, remind them of deadlines, and encourage them to complete the tasks on time.

### Quantitative analysis

In the 2022–2023 academic year, 104 parents consented to participate in the Parent University program at our university. We randomly assigned 52 parents to the Control Group and 52 parents to the Intervention Group. In the following 2023–2024 academic year, another 111 parents consented to participate in the program. We randomly assigned 56 parents to the Control Group and 55 to the Intervention Group. Each year, we conducted a survey to evaluate parents’ engagement at the beginning of the academic year (i.e., before the intervention). At the end of the academic year, we conducted a second survey (i.e., after the intervention) to investigate potential changes in engagement and feelings about the program. The surveys were sent to parents in the Control Group and the Intervention Group. The surveys gathered information regarding parents’ demographic background and their interactions/relationships with their children enrolled at the college. Due to the small number of parents completing both surveys (i.e., the survey before the intervention and the survey after the intervention), we combined the data collected from the years 2022–2023 and 2023–2024.

Table 2 displays the descriptive results. There were 27 parents in the intervention group and 32 parents in the control group. The majority of the parents identified themselves as mothers of the students (89% in the intervention group and 91% in the control group). The majority of parents identified as Black/African American (85% in the intervention group and 81% in the control group). The average age of parents was 48 (SD = 8) in the intervention group and 45 (SD = 6) in the control group. Fifty-six percent of the parents were married in the intervention group and 42% in the control group. The majority of parents were employed (including full-time, part-time, and self-employed) at the time of the survey (93% in the intervention group and 91% in the control group). It should be noted that the majority of parents indicated that they had at least a Bachelor’s degree (74% in the intervention group and 66% in the control group).

The pre-and post-surveys asked questions regarding how worried they were about their child in college in terms of academic, social, health, and career factors at the time of completing the survey. There were 17 items for worriedness ratings to reflect areas of parental Concerns^1^, including “under too much academic stress,” “lonely or isolated,” “may drink too much,” “not being prepared for career,” “made wrong friends,” “will choose the wrong career,” “not performing up to their abilities,” “not eating right,” “under too much peer pressure,” “not performing at the top of their class,” “not able to manage health issues,” “not safe from crime,” “not studying enough,” “not attending religious services,” “not making academic progress,” “not exercising,” and “trouble finding a job after graduation.” Parents could rate their worriedness level for each item as “Not at all,” “Somewhat,” “Quite a bit,” and “A great deal.” We combined “Not at all” and “Somewhat” into one category “low level” and coded 0. We also combined “Quite a bit” and “A great deal” into one category “high level” and coded 1. It should be noted that the questions were not previously tested and validated. We chose these questions because they reflect the goals of the study and collect critical data for our evaluation. In addition, to our knowledge, there is no validated questionnaire available on college parents’ worriedness and engagement in the U.S. This further raises the importance of the current study.

For the intervention group, the percentages of parents reporting a high level of worriedness for “may drink too much,” “not perform at the top of their class,” and “not attending religious services” decreased from the pre-intervention survey to the post-intervention survey. However, worriedness level increased for many items, including “under too much academic stress,” “lonely or isolated,” made wrong friends,” “will choose the wrong career,” “not performing up to their abilities,” “under too much peer pressure,” not safe from crime,” “not studying enough,” “not making academic progress,” and “not exercise.”

We further constructed a score using the 17 items to determine overall worriedness. We added all 17 items (for each item, 0 represents a low level of worriedness and 1 represents a high level of worriedness). After adding all items, the constructed score of worriedness ranged from 0 to 17, 0 representing the lowest level of overall worriedness and 17 representing the highest level of overall worriedness. For the intervention group, the average score of worriedness was 1.26 (SD = 1.58) before the intervention and 2.26 (SD = 1.46) after the intervention. For the control group, the score of worriedness also went up, although slightly, i.e., 2.43 (SD = 3.26) before the intervention and 2.75 (SD = 3.13) after the intervention.

On both surveys, we asked parents to rate their level of engagement with their child’s college education and career development. The scores ranged from 1 to 10, with 1 representing the lowest level of engagement and 10 representing the highest level of engagement. The average parental engagement for career development was 7.78 (SD = 2.59) before the intervention and 7.67 (SD = 2.43) after the intervention, decreasing slightly. Parental engagement in college education also decreased, from 8.33 (SD = 2.13) before the intervention to 8.18 (SD = 2.20) after the intervention. For the control group, however, the results showed the opposite trend. In control participants, average parental engagement for career development increased from 7.38 (SD = 2.42) before the intervention to 8.18 (SD = 2.21) after the intervention. In addition, the average parental engagement for college education went up from 8.28 (SD = 1.94) before the intervention to 8.63 (SD = 1.64) after the intervention.

We further divided our sample into groups based on whether the parents received a bachelor’s degree or not. Table 3 summarizes descriptive results for parents who were not college educated^2^ (8 parents in the intervention group and 11 parents in the control group). The majority of these participants who were not college-educated were mothers. The average age was 50.5 (SD = 10.76) in the intervention group and 41.91 (SD = 5.68) in the control group. In addition, the majority of these parents indicated they were employed at the time of the survey (75% in the intervention group and 91% in the control group). In the intervention group, 62.5% of the parents indicated that they were married. This number was only 9.09% in the control group.

In terms of the constructed score of worriedness for parents without college education, the average number was 1.63 (SD = 2.33) before intervention and 2.12 (SD = 1.96) after intervention among the parents in the intervention group. The trend is similar in the control group. The number went from 2.62 (SD = 3.83) in the pre-intervention survey to 3.27 (SD = 4.15) in the post-intervention survey.

Similar trends were found for parental engagement among parents who were not college educated. For example, in the intervention group, the average reported parental engagement for career development decreased from 8.38 (SD = 2.50) before the intervention to 8.13 (SD = 3.09) after the intervention. In addition, parental engagement for college education decreased from 9.13 (SD = 1.81) before the intervention to 8.75 (SD = 2.12) after the intervention. The opposite trend was observed for the control group. In the control group, parental engagement for career development increased from 6.64 (SD = 2.98) in the pre-intervention survey to 8.36 (SD = 2.5) in the post-intervention survey. Likewise, parental engagement for college education increased from 8.45 (SD = 1.75) in the pre-intervention survey to 9.09 (SD = 1.38) in the post-intervention survey.

Table 4 displays results from a paired-sample t-test analysis on worriedness level, as well as parental engagement in career development and college education^3^. Two significant results were found. In the intervention group, the *t*-test result revealed a significant difference in worriedness level between pre-and post-intervention surveys (t = 1.9640, *p* = 0.0302), indicating that there was a significant increase in parents’ level of worriedness compared to the pre-intervention level. In the control group, the t-test revealed a significant increase in parental engagement for career development across the pre-and post-intervention surveys (t = 1.9352, *p* = 0.0311), indicating enhanced engagement at the end of the freshman year compared to the beginning of the freshman year.

Table 5 displays results from the t-test analysis on several outcome measures, including worriedness, parental engagement for career development, and parental engagement for college education by groups and by parental education. Results indicated that among parents who were college educated in the intervention group, the t-test result was significant (t = 2.0011, *p* = 0.0303) for worriedness level, indicating increased worriedness at the end of the freshman year compared to the beginning of the freshman year. However, in the control group, parents without a college education showed a significant increase in parental engagement for career development (t = 1.7593, *p* = 0.0531) at the end of the freshman year compared to the beginning of the freshman year.

### Qualitative analysis

Some results from the quantitative surveys were unexpected. For example, the quantitative results suggested that, following the intervention, parents in the intervention group showed increased worriedness level compared to pre-intervention levels. In addition, there was a significant increase in parental engagement for career development among parents in the control group, but not in the intervention group. To further investigate the quantitative findings, we conducted in-depth interviews with 7 parents in the intervention group to learn more about their experiences helping their children navigate college. The interviews were semi-structured. Some questions were prepared beforehand. The main questions included how often they communicated with their child (and what topics they discussed), how would they describe their engagement level at the beginning and at the end of the freshman year, how was the child’s transition from high school to college, and what challenges did the parents face in helping their child navigate college. Several themes emerged from these interviews.

### Theme 1: parental engagement levels decreased as time went on

The interviews helped us understand why we observed decreased engagement in the surveys. In the interviews, many parents indicated that as their child grew more independent in college, the parents learned to trust their children more. Therefore, compared to the beginning of the freshman year, the parents were not as engaged nor did they spend as much time searching for resources. In addition, they reported that the information they acquired from Parent University was useful in helping them guide their child. Parents spent less time searching for information online or trying to figure out how to help their child, because the Parent University program provided the information. For example:

At the beginning, I think I was maybe a little bit more hands on just to try to make sure that she knew you need to be on time, you know, get up for your classes. Just trying to build a structure for her, and then after that I feel like I just it was more hands off. [I] just to allow her just like check through information getting from parent university following up with her, just to make sure that she was staying focused. So I feel like initially it was a lot of hands on just trying to make sure that she stayed focused, and then just kind of letting go for her to just do her own thing.

### Theme: 2: parents with college experience still need informational guidance

Although many parents in our program were college educated, they all recognized that things have changed since they went to college. They acknowledged that without the information from Parent University, it would have been hard for them to help their child navigate college. This is consistent with the quantitative findings. For example, among parents who were college-educated in the intervention group, their worriedness level increased at the end of the freshman year compared to the beginning of the freshman year.

I think my college education certainly prepared me for a lot of things in my career, but as a parent, my experience with college … Just my experience being in college and kind of knowing what to expect. Obviously, things have changed in over 30 years. Things have changed.

### Theme: 3: a parent university program tailored to child’s year in college (i.e., freshman to senior)

Many parents expressed that a Parent University program tailored to their child’s year in college (i.e., freshman, sophomore, junior, or senior) would be exceptionally helpful. For example, many of their children are now sophomores and juniors. Parents would like more information about internships and job opportunities, so they can continue to help their children with new challenges that typical arise after freshman year.

I wish that [the Parent University Program] would continue because yes, we learn stuff [during the] freshman year. But, sophomore year … You know, I want to make sure that my daughter is staying on track. So, what do I need to think about freshman year? I wasn’t thinking about internships. I’m thinking about just our success and acclimation to the university as a freshman, but now I need to start thinking, OK? Well, what is she going to do this summer?

### Theme: 4: parent university assisted parents in helping their children to navigate college

All parents expressed during the interview that the Parent University program was very useful in helping their child navigate college and that parents successfully communicated the information to their children. This result is consistent with the quantitative findings.

I feel like that this Parent University is something that all parents, whether you went to college or not, can help keep focused because we do try to do well, you know, college is set up for your child to be successful.

So, I do like that there was a parent engagement forum for parents of [the university], you know, freshmen to be able to get acquaintance to the university. And what steps parents could take to support our children because I didn’t have that.

### Summary and conclusion

Parental educational level plays an important role in children’s educational outcomes, including college success (Coleman, 1968; Belzil and Hansen, 2003). Intergenerational transmission of education is regarded as one of the central mechanisms underlying educational inequality. One way to measure intergenerational educational mobility is to examine the association between the parent’s and child’s schooling (Andrade and Thomsen, 2018). A high degree of intergenerational transmission of education indicates that parental educational background plays an essential role in children’s education. The intergenerational educational mobility remains low in the U.S. Therefore, first-generation students from underrepresented minority backgrounds might not have the cultural capital from parents to help them navigate college (Wang and Sakamoto, 2021). Underrepresented minority students are less likely to go to college, more likely to drop out of college, and are less likely to complete college in 4 years. Even for minority students whose parents went to college, research has shown that White Americans leverage their parental education advantages at higher rates than Black Americans (Long et al., 2012). For example, one study found that the benefits of parental education on child’s educational outcomes are smaller among Black students compared to White students especially in urban areas (Assari et al., 2021). In other words, Black Americans might not experience the same level of benefit from parental education compared to White Americans even among those parents who went to college. The Parent University program at our HBCU aimed to empower parents with the information they needed to help their children navigate college life. The Parent University program involved working with multiple university offices and personnel to impart important information to parents. The information included but was not limited to resources on academic life, internships and research opportunities, financial aid and loans, campus resources, health resources, and residential life. The information was shared with parents through virtual live forums, recordings of the forums, as well as text messages and emails. The overarching goal of Parent University was to equip parents with knowledge that they can use to guide their children toward a successful college career.

Despite the unique challenges of implementing the Parent University program, including the COVID-19 pandemic, the Parent University program recruited 3 cohorts of parents, with one cohort being the pilot cohort. The majority of parents in the program indicated that the program was helpful and that they successfully communicated the information to their children. Our quantitative and qualitative analyses further suggested that parents in the intervention group reported lower levels of effort helping their child navigate college, demonstrating the benefits of the Parent University Forums and other shared resources.

In addition, our implementation of the Parent University project revealed some best practices for conveying information to parents, as well as some areas that require further improvement. For example, we found that communication was most effective using text messages delivered directly to the parents’ cell phones. Other methods were not as effective. For instance, we worked with IT services on our campus to give parents access to the University’s Blackboard learning management system and email addresses affiliated with our university, so they could receive emails and BlackBoard announcements. However, we found that parents did not check their university emails or Blackboard announcements on a regular basis. In addition, the response rate to the surveys remained low across the three cohorts (i.e., *n* = 27 intervention, n = 32 control), despite efforts to reach out to the parents, multiple reminders, and offering monetary compensation for completing the surveys. The low survey response rates created some challenges for the program evaluation. For example, the survey respondents might not be a representative sample of the parents who participated in the program; however, the results still represent an important first step in understanding the effectiveness of the program. To further evaluate Parent University and similar programs, investigators should develop and test innovative strategies to enhance survey response rate (i.e., by using automated reminders or additional incentives).

## Figures and Tables

**TABLE 1:** Parent university forum topics.

Forum topic	Description	Department
Parent University Program Orientation	The PU Program Team reviews the program and the role of participants with the parents.	The Parent University Team
Next Steps for Next Semester	It discusses what students need to do to be prepared for the spring semester. Pre-registration, satisfied student accounts, deadlines and the importance of meeting pre-requisite requirements will be addressed. Also, academic advisement, curriculum guides, early alerts, satisfactory academic progress (SAP), senior audits, and other topics that pertain to student success will be addressed.	The Offices of Records, Registration and Student Success
Preparation to Transition from the Classroom to the Workplace	Applications to participate in co-curricular learning experiences (i.e., internships, cooperative education programs, study abroad excursions, on campus student employment etc.) and scholarships need to be submitted very early in the spring semester. Parents will learn about these opportunities and what students need to do to take advantage of them.	Career Center
How Are Your Student’s Affairs?	Parents will have an opportunity to hear from and engage University representatives that manage student life on campus	Residence Life and Student Leadership and Engagement
Helping Hornets Stay Healthy and Safe	It will share the services and programming provided for students and a representative from the Campus Police Department will also be available to address security alerts and provide any information or updates that parents need to be aware of regarding campus safety	The Office of Counseling Services, the Wellness and Recreation Center, and The Campus Police Department
Getting a Jump on Fall	Tips for getting your students advised, registered, housed, and paid up for the fall semester.	Offices of Student Success, Financial Services, Resident Life, Records and Registration, Student Accounts, and Student Health Services
Parent University Program Closing Session	This forum will be an open discussion that provides an opportunity for participants to share and provide measurable feedback on the Parent University Program	The Parent University Team

**TABLE 2:** Descriptive statistics: the whole sample.

Variable	Intervention group		Control group	
	Before	After	Before	After
N	27	27	32	32
Mother %	88.89		90.62	
Black %	85.19		81.25	
Age average	47.74 (SD = 7.93)		45.31 (SD = 5.74)	
Married %	55.56		41.94	
Employed %	92.59		90.62	
> = BA degree %	74.07		65.62	
Frequency communicate with child %				
More than once a day	25.93	33.33	9.38	25.00
Daily	48.15	33.33	46.88	37.50
Weekly	25.92	33.33	3.12	37.50
Other	0	0	3.12	0
Total	100	100	100	100
Worried my child in college (is)…… %				
Under too much academic stress	11.11	25.92	25.00	28.12
Lonely or isolated	18.52	25.92	21.88	31.26
May drink too much	7.41	0	3.12	3.12
Not being prepared for career	7.40	7.40	12.50	15.63
Made wrong friends	0	7.400	9.37	9.37
will choose the wrong career	0	3.7	3.12	0
not performing up to their abilities	0	18.52	9.37	15.63
not eating right	22.22	25.92	25.00	43.76
under too much peer pressure	0	14.82	12.50	12.50
not performing at the top of their class	14.81	7.41	9.37	12.50
not able to manage health issues	3.70	3.70	9.38	3.12
not safe from crime	11.11	40.74	25.00	43.75
not studying enough	7.40	18.51	15.63	15.63
not attending religious services	15.11	7.41	9.37	12.50
not making academic progress	0	11.11	9.37	15.63
not exercise	0	14.81	25	12.50
trouble finding a job after graduation	7.40	7.40	18.75	0
Constructed score of worriedness	1.26 (SD = 1.58)	2.26 (SD = 1.46)	2.43 (SD = 3.26)	2.75 (SD = 3.13)
Communicated PU information to child		77.78		NA
Knows GPA %		70.37		62.50
GPA average		3.04 (SD = 1.10)		3.49 (SD = 0.57)
Parental engagement career development	7.78 (SD = 2.59)	7.67 (SD = 2.43)	7.38 (SD = 2.42)	8.18 (SD = 2.21)
Parental engagement college education	8.33 (SD = 2.13)	8.18 (SD = 2.20)	8.28 (SD = 1.94)	8.63 (SD = 1.64)

**TABLE 3:** Descriptive statistics: parents without a college degree.

Variable	Intervention group		Control group	
	Before	After	Before	After
N	8	8	11	11
Mother %	87.50		100.00	
Black %	75.00		81.82	
Age average	50.50 (SD = 10.76)		41.91 (SD = 5.68)	
Married %	62.50		9.09	
Employed %	75.00		90.91	
Frequency communicate with child %				
More than once a day	25.00		27.27	
Daily	62.50		45.45	
Weekly	12.50		27.27	
Other	0		0	
Total	100		100	
Worried my child in college (is)…… %				
under too much academic stress	12.50	37.50	27.27	27.27
lonely or isolated	12.50	25.00	36.36	18.18
may drink too much	0	0	0	0
not being prepared for career	0	0	9.09	27.27
made wrong friends	0	0	9.09	9.09
will choose the wrong career	0	0	9.09	0
not performing up to their abilities	0	25.00	18.18	18.18
not eating right	50.00	37.50	18.18	36.36
under too much peer pressure	0	0	18.18	27.27
not performing at the top of their class	12.50	12.50	9.09	18.18
not able to manage health issues	12.50	0	0	0
not safe from crime	25.00	37.50	27.27	45.45
not studying enough	0	12.50	18.18	18.18
not attending religious services	12.50	12.50	9.09	9.09
not making academic progress	0	0	9.09	27.27
not exercise	0	12.50	18.18	18.18
trouble finding a job after graduation	12.50	0	27.27	0
Constructed score of worriedness	1.63 (SD = 2.33)	2.12 (SD = 1.96)	2.63 (SD = 3.83)	3.27 (SD = 4.15)
Communicated PU information to child		75.00		
Knows GPA %		50.00		36.36
GPA average		3.20 (SD = 0.54)		3.68 (SD = 0.14)
Parental engagement career development	8.38 (SD = 2.50)	8.13 (SD = 3.09)	6.64 (SD = 2.98)	8.36 (SD = 2.50)
Parental engagement college education	9.13 (SD = 1.81)	8.75 (SD = 2.12)	8.45 (SD-1.75)	9.09 (SD = 1.38)

**TABLE 4:** Results from paired *t*-test analysis: the whole sample.

Variable	Intervention group	Control group
	**t = 1.9640**	t = 0.7124
	**p = 0.0302**	*p* = 0.2408
	t = −0.2166	**t = 1.9352**
Level of worriedness	*p* = 0.5849	**p = 0.0311**
	t = −0.7498	t = 0.9524
Parental engagement college education	*p* = 0.7699	*p* = 0.1741
N	27	32
df	26	31

Bold values indicate *p* <= 0.05 level.

**TABLE 5:** Results from paired *t*-test analysis by parental education.

Variable	Intervention group		Control group	
	<BA	> = BA	<BA	> = BA
Level of worriedness	t = 0.5092	**t = 2.0011**	t = 0.6916	t = 0.3541
	*p* = 0.3131	**p = 0.0303**	*p* = 0.2518	*p* = 0.3636
Parental engagement career development	t = −0.1839	t = −0.1082	**t = 1.7593**	t = 0.9073
	*p* = 0.5703	*p* = 0.5425	**p = 0.0531**	*p* = 0.1878
Parental engagement college education	t = −0.8143	t = −0.2518	t = 1.3582	t = 0.2104
	*p* = 0.7789	*p* = 0.5980	*p* = 0.1008	*p* = 0.4178
N	8	19	12	20
df	7	18	11	19

Bold values indicate *p* <= 0.05 level.

## Data Availability

The raw data supporting the conclusions of this article will be made available by the authors, without undue reservation.

## Footnotes

1 The items were adapted from questions in The Parent Survey (The Office of Institutional Effectiveness, Research and Assessment, Swarthmore College, 2007).

2 Results among parents who had a college degree are available upon request.

3 The primary goal of this project was to evaluate whether there was a statistically significant change in the measures pre-and post-intervention, rather than to estimate the magnitude of the effect. Therefore, we reported the t test value, *p* value, and the degrees of freedom. Cohen’s d values are available upon request.
